# Mechanistic basis for morphological damage induced by essential oil from Brazilian pepper tree, *Schinus terebinthifolia,* on larvae of *Stegomyia aegypti*, the dengue vector

**DOI:** 10.1186/s13071-015-0746-0

**Published:** 2015-03-01

**Authors:** Drielle L A Pratti, Alessandro C Ramos, Rodrigo Scherer, Zilma M A Cruz, Ary G Silva

**Affiliations:** Programa de Pós-graduação em Ciências Farmacêuticas, Universidade Vila Velha – UVV, Rua Comissário José Dantas de Melo, 21, Boa Vista, Vila Velha, ES Brazil; Programa de Pós-graduação em Ecologia de Ecossistemas, Universidade Vila Velha – UVV, Rua Comissário José Dantas de Melo, 21, Boa Vista, Vila Velha, ES Brazil; Tommasi Analítica, Avenida Luciano das Neves, 2016, Divino Espírito Santo, Vila Velha, ES Brazil

**Keywords:** Aroeira, Christmas berry, Larvicide, *Aedes*, Cullicidae, Anacardiaceae

## Abstract

**Background:**

Dengue has become the subject of public health programs worldwide. The lack of a vaccine and the high environmental risk of synthetic insecticides, arouse the interest in natural products against this vector. This study aimed to determine the chemical composition of the essential oil of ripe fruits and seeds of *Schinus terebinthifolia* Raddi; to evaluate the essential oil effect on mortality of *Stegomyia aegypti* (Linnaeus, 1792) larvae; and to characterize the structural damage suffered by larvae and their association with different contents of essential oil.

**Methods:**

Ripe fruits and seeds were crunched and their essential oil was extracted through hydrodistillation, purified, and its phytochemical analysis was carried out through High Resolution Gas Chromatography, coupled with Mass Spectrometry. This essential oil was diluted in a 10-point gradient of 86.22 – 862.20 ppm, at regular intervals of 86.22 ppm. Each point received 50 larvae and the assessments of surviving were made at 24, 48 and 72 hours after inoculation. Structural damage was assessed through measurements of thickness with exoskeleton, evaluating the integrity of the head, thorax, abdominal segments, and air siphon, using ImageJ® software. Statistical data analysis was carried out through Logistic Regression and Discriminant Analysis.

**Results:**

56 substances were identified, corresponding to 81.67% of the essential oil composition. Larvae were dose-dependent susceptible to the essential oil; the concentration produced a significant effect on larval mortality. Among the major deformations found in the larvae, it was detected inhibition of chitin synthesis by the activity of the oil, thus reducing the deposition of cuticle layers.

**Conclusion:**

The essential oil caused death in exposed larvae after 72 hours, in a dose-dependent manner. It also changed the structure of exposed larvae, indicating a direct effect on larval exoskeleton. The results open up possibilities for the use of natural products as an alternative to control dipterans.

## Background

The expansion of air transportation and maritime commerce overcome geographical barriers for insect vectors of disease, allowing them to cover large distribution areas in short periods of time. When transportation was limited to going on foot or animals, the diseases were confined to particular regions of the world [1]. With the growing movement of large numbers of people by land, sea and air, multiple infections were spread worldwide. With the advent of boats and the emergence of intercontinental travel during the initial planet explorations, these infections have spread geographically. They had reached regions isolated from each other [2], representing typical cases of bioinvasion [3].

Among the diseases spread by the modernization of the transportation network, yellow fever and dengue are essential to global public health. They have a common mosquito vector, the *Stegomyia aegypti* (Linnaeus, 1762). It is native to tropical and subtropical West Africa, which have spread and adapted well to anthropic and outdoor breeding sites, as well as to water storage containers on ships [4]. In this way, with the Great Navigations and the slave trade, this species arrived in Portugal and Spain, especially in harbor cities. It also established in all temperate and tropical regions of the Americas, with European colonization of the New World, causing major epidemics [5].

The first reports on the presence of *St. aegypti* on Brazilian territory dates back to 1686. It was associated with an epidemic of yellow fever in Bahia State, despite the previous epidemic in 1685 in Recife, for which there were no explicit records of that vector [2]. This vector was supposedly eradicated from Brazil in 1955 [6]. However, new outbreaks were reported in the 70’s. In current times of climate change, dengue has become a pandemic of high importance to public health. It is due to the propagation of the mosquito vector throughout the intertropical region that has the highest population densities on the planet [7].

With the increasing spread of dengue, it is expected to make a large impact on the global health sector, and consequently in the world global economy. Every year, an average of 574, 000 cases are reported, representing a total annual estimated cost of at least I$ 587 million in the eight countries. This value can increase to US$ 1.8 billion if we consider the probable case is underreporting. When the costs of surveillance and vector control are added, the final amount will be even higher [8].

There is still no vaccine against dengue, development has been hampered by the existence of four viral types [9]. The most efficient preventive action has been the control of mosquito vector populations in the adult stage; human populations are vulnerable to epidemics. The emergence of epidemics, such as dengue, has been associated with a high level of human population density and unplanned urbanization [10], and with the high domestication of its vector [11]. The mosquito control has been done mainly with the use of organophosphate and pyrethroid insecticides [12]. Between the years 1940 to 1980, those substances were widely used without restrictions and with great success. However, throughout the years, studies have shown that mosquitoes acquired some resistance to these insecticides [12,13], which also have produced large damage to ecosystems [14,15]. Thus, the search for new ways of vector control had started, including natural products, given the high diversity of plants, particularly in the tropics where dengue epidemics have been concentrated [16].

Besides that, the most appropriate strategy for the control of populations of the mosquito vector is still controversial. Despite the elimination of adult as the most efficient measure in combating dengue, several studies have been conducted to investigate the performance of both natural and synthetic substances, with ovicidal and larvicidal activities [17]. Regarding the larvicidal activity, natural products have high potential as described by Chowdhury et al. [18], who worked with aqueous extracts of *Solanum villosum* Mill. (Solanaceae) and by Silva et al. [16], which examined the essential oil of *Schinus terebinthifolia* Raddi (Anacardiaceae). Both studies attained satisfactory results as to the larvicidal activity against *St. aegypti*.

*Schinus terebinthifolia* Raddi, also known as aroeira, the Brazilian pepper tree, belongs to the family Anacardiaceae [9,19]. It is a perennial tree, native to South America, found in Brazil, Paraguay, and Argentina [20]. In Brazil, the dried ripe fruits of *S. terebinthifolia* are marketed as a substitute for black pepper. Many medicinal properties have been attached to this plant, such as antioxidant, wound healing, antitumor and antimicrobial activities [21] and larvicidal activity against Dengue mosquitoes, *St. aegypti* [16]. Considering the already proven larvicidal activity of this essential oil [16,20], the present study had evaluated the structural damages induced in *St. aegypti* larvae exposed to it. In parallel, we determined the qualitative and quantitative chemical composition of the essential oil of ripe fruits and seeds of *S. terebinthifolia*; evaluated its activity on the *St. aegypti* larval mortality; and investigated their correlation with different concentrations of essential oil to which the larvae were exposed.

## Methods

### Plant material and essential oil characterization [deposition of reference samples]

Ripe fruits and seeds of Brazilian pepper were harvested in the region of Vitória, Espírito Santo State, at the geographical coordinates 20°19’36 - 20°19’46”S, and 40°16’38”W. Vouchers of fruity plant material were deposited at the Herbarium of the Universidade Vila Velha, under registry number UVVES-2205. Fruits and seeds were cleaned, freed from impurities, and divided into ten samples of 100 g each. Fruits were washed with deionized water and placed in round bottom flask [22] for extraction in Clevenger apparatus, for four hours after water boiling for each sample. Heating was maintained at the minimum temperature required for boiling. The entire extraction process was carried out in the Laboratory of Functional Ecology of UVV. After extraction, samples were poured into glass flasks, and the purification was performed by freezing the remaining water. The density of each essential oil sample was determined gravimetrically by weighing 1 mL liquid at 20°C on an analytical balance, accurate to 1.0 mg, in a temperature-controlled room.

The chromatographic analysis of essential oil components was performed by high-resolution gas chromatography, coupled to mass spectrometry (GC-MS). The injection volume was 2 μL, made up of 1.8 μL essential oil (30 mg/ml) and 0.2 μL solution of a series of C7-C30 hydrocarbons, as an internal standard in *n*-hexane. The system used in CG-EM consisted of a gas chromatograph, Ultra GC Thermo Scientific® coupled to a mass spectrometer Thermo Scientific®. The stationary phase of the chromatographic column was fused silica (DB-5 J & W Scientific, 30 m × 0.25 mm × 0.25 mm). Helium was the carrier gas, and the column temperature was increased by 3°C per minute from 60° to 240°C. Mass spectra were obtained at 70 eV with a scan rate of 0.84 scan/sec, in the m/z range of 40–500 [23]. The retention time of sample components and a mixture of n-alkanes of C7-C30, co-injected into the GC-MS system at the same temperature program, were used to calculate the Kovats Retention Index - KI [23] and the van Den Dool and Kratz retention index 1963 [24].

The files with the mass spectra obtained from the scans were used to identify the components of the essential oils, in the Laboratory of Functional Ecology of UVV, by using the Xcalibur software, version 2.0.7. The identification was based on the spectral similarity made by comparing the spectra obtained with those in the spectral library and literature available, and comparing the retention indices calculated with those available in the literature [23].

### Larval bioassay

Third stage larvae of *St. aegypti*, obtained by incubating eggs in 3 L natural water and supplying food for fish, Krill Tropical Flakes® were used in bioassays. Eggs were collected by ovitraps, consisting of black plastic containers filled with water up to half of its capacity, and provided with Eucatex® palettes for egg attachment. Ovitraps were placed in the facilities of the University Vila Velha, near the trees and plants on the campus.

We prepared a series of tubes with five replicates for the blank control and each treatment, and each replicate received 10 live larvae. The blank control was used to evaluate the survival of larvae in a solution of Tween® 80 at 0.5% in deionized water.

Silva et al. (2010) reported total mortality of larvae exposed to essential oil at 862.20 ppm [16]. Thus, we prepared a concentration gradient between 86.22 – 862.20 ppm of essential oil, dispersed in a solution of 0.5% Tween® 80. Ten dilutions were made at 86.22 ppm intervals, taking the upward vertical migration of larvae for breathing as a survival criterion. Assessments of surviving larvae were made at 24, 48 and 72 hours after inoculation with safety to prevent the emergence of adult mosquitoes.

### Morphometric analysis of larvae

Larvae were fixed with a mixture of formaldehyde, acetic acid, ethanol and water (4:3:50:43). They were mounted on glass slides and photographed under magnification (Leica Zoom® 2000) with a graphic scale of 3000 μm. Thickness measurements with exoskeleton projection were made of the eight segments of the body of larvae using the larval digestive tract as an internal reference, were made using ImageJ® software. We examined the integrity of the head, thorax, abdomen, and air siphon.

Among a total of 550 larvae distributed in the gradient of essential oil concentrations up to 689.76 ppm, 247 larvae that had some structural damage were observed under magnification (Leica Zoom® 2000).

### Statistical analysis

The chemical diversity of the 10 samples of essential oil was estimated by the Shannon-Weaver Diversity Index (H'). Their evenness was assessed by Pielou index *(J*) [25], and both were tested for possible differences by *t*-test for independent samples. The significance level for rejecting the null hypothesis of equality of the mean indices was *p* < 0.05 [26].

The percentage of larval death for each replication was transformed to the arcsine of its square root. Their normality was verified by a K^2^ test, based on deviations from symmetry and kurtosis of the probability curve of data distribution relative to the null hypothesis of a normal distribution. Another assumption for parametric tests, homoscedasticity was confirmed by Bartlett test [26].

Since mortality rates did not meet the normality assumption, a binary logistic regression was run, based on a probit model. Doses and duration of exposure to essential oil were the independent variables, and larval mortality was the dependent one. The null hypothesis tested was that the larval mortality rate was independent of dose or exposure time to the essential oil through a probit model based on the neperian logarithmic transformation [27]. Lethal concentrations, LC_50_, LC_90_, LC_95_, and LC_99_ were calculated from the line equation generated by the probit model tested [27]. Statistical analyzes were carried out using the softwares Systat 13.0 and Minitab 17.0.

Structural damages in larvae were evaluated by a Discriminant Analysis to check for differentiation of morphological groups consistent with the different concentrations of the essential oil to which they were exposed. The consistency of the estimated discriminant function was verified by the cross-validation method of Jackknife [28]. The Discriminant Analysis was run in the software Systat 13.0. The association between the major components of the essential oil on larval damages was tested by an ordinal logistic regression, testing the hypothesis that the major compounds in the essential oil cause the observed structural damage. The level of significance was set at *p* < 0.05 [27].

## Results and discussion

### Essential oil

The essential oil extracted from ripe fruit was presented as a low viscosity liquid, colorless and translucent. Considering the mean values and their respective 95% confidence intervals (CI), the extraction yield of essential oil of ripe fruits and seeds was 2.729 ± 0.253%, absolute density was 0.8622 ± 0.002 g.mL^−1^; the chemical diversity (*H'*) was 2.057 ± 0.147; and evenness (*J*) was 0.487 ± 0.030.

Among the 56 substances identified in the essential oil, mainly mono- and sesquiterpenes, the major compounds were δ-3-carene, 55.36%, α-pinene, 15.62%, and sylvestrene, 10.69%, which corresponded to 81.67% of the total oil composition. When the 12 minor components that comprise 14.44% were considered, 96.11% of the total essential oil composition was detected and identified. Some of the components were sesquiterpenoids and phenylpropanoids, in addition to the monoterpenes above (Table 1). The other 41 compounds were present in residual traces and formed 3.89% of the total composition.

**Table 1:** **Major components of the essential oil of ripe fruits and seeds of** ***Schinus terebinthifolia*** **Raddi**

**Retention indices**				**Identification %**	
**van den Dool and Kratz**		**Kovats**			
**Calculated**	**Adams 2009**	**Calculated**	**Adams 2009**		
1010	1008	1011	1011	δ-3-carene	55.36
933	932	938	939	α-pinene	15.62
1027	1025	1031	1030	sylvestrene	10.69
1479	1480	1481	1481	germacrene D	2.48
1377	1379	1379	1381	β-patchoulene	1.99
988	988	990	990	mircene	1.89
1355	1356	1357	1359	eugenol	1.71
1087	1086	1089	1088	terpinolene	1.52
1392	1390	1393	1391	sativene	0.93
1418	1419	1419	1420	β-cedrene	0.89
1675	1674	1676	1675	cis-α-santalol	0.77
1447	1448	1449	1450	cis-muurola-3,5-diene	0.76
1546	1546	1548	1548	hedycaryol	0.66
1336	1335	1339	1338	δ-elemene	0.44
1434	1434	1436	1436	γ-elemene	0.41
			Total		96.11

The evenness illustrates the uniformity in distribution of proportions of chemical substances into the species studied, indicating the degree of symmetry in the proportional distribution of mass between the components of essential oils. Values less than 1 indicate an increasingly uneven concentration of the components as they approach zero, which is very common in essential oils in which more than 40 substances are easily identified, but only a small number is responsible for the higher proportion of mass [29].

Essential oils can be present in a single organ or the whole plant [22]. They can be produced in secretory cells, cavities, ducts, epidermal cells and trichomes and have a lower density than water, also evidenced for the studied essential oil, being soluble in organic compounds. They usually contain about 20–60 substances at different concentrations, and in general 2 or 3 of these substances have higher concentrations (20 - 70%) and determine their biological property [29].

Qualitative and quantitative changes may occur in the composition of essential oil from the same plant species. These differences may result from intraclonal variation in breeding cultivars with vegetative propagation of rose-scented geranium, *Pelargonium* sp., suggesting that even somatic genetic variations can affect the biosynthesis and production of essential oil [30].

These differences may also be due to environmental causes. Among them are the geographical origin, stress by abiotic factors such as wind, humidity, and salinity [31]. The cultivation form can also induce variations in the essential oil composition [13]. However, as it is a material obtained from species growing under natural conditions, it is likely that the differences detected are derived from differences in geographic location of the plant from which the essential oil was extracted.

### Larval bioassay

No larval mortality was detected in the blank control (Table 2), indicating the negligible effect of the diluent solution with Tween 80® on larval mortality. No larvae survived at the concentration of 862.20 ppm (Table 2) or higher concentrations, dying within one hour after exposure to those essential oil concentrations.

**Table 2:** **Mortality of larvae of** ***Stegomyia aegypti*** **(Linnaeus, 1792) after exposure to a concentration gradient of the essential oil of ripe fruits and seeds of** ***Schinus terebinthifolia*** **Raddi**

**Essential oil (ppm)**	**Larval mortality (% Mean ± standard error)**		
	**24 h**	**48 h**	**72 h**
Blank	0.00 ± 0.00	0.00 ± 4.00	0.00 ± 4.00
86.22	0.00 ± 0.00	2.00 ± 2.00	4.00 ± 2.45
172.44	42.00 ± 22.00	48.00 ± 19.85	52.00 ± 18.00
258.66	26.00 ± 19.39	32.00 ± 17.45	32.00 ± 22.00
344.88	24.00 ± 12.08	38.00 ± 14.97	42.00 ± 18.85
431.10	74.00 ± 14.00	82.00 ± 9.17	88.00 ± 8.00
517.32	60.00 ± 24.49	64.00 ± 22.27	64.00 ± 22.27
603.54	60.00 ± 24.49	62.00 ± 23.32	64.00 ± 22.27
689.76	62.00 ± 23.32	64.00 ± 22.27	68.00 ± 19.85
775.98	80.00 ± 20.00	80.00 ± 20.00	82.00 ± 18.00
862.20	100.00 ± 0.00	100.00 ± 0.00	100.00 ± 0.00

Larval mortality was not exclusively attributed to the essential oil concentration in the incubation medium. Exposure time to the essential oil also affected the mortality rate significantly (Table 3).

**Table 3:** **Binary logistic regression between concentration and exposure time to the essential oil of ripe fruits and seeds of** ***Schinus terebinthifolia*** **Raddi and larval mortality of larvae of** ***Stegomyia aegypti*** **(Linnaeus, 1792)**

**Parameter**	**Parameters**			
	**Coefficient**	**EP**	**Z**	***p***
Essential oil concentration (PPM)				
Constant	−1.086	0.069	−15.81	<0.01^hs^
PPM	0.003	0.0001	17.30	<0.01h^s^
G = 330.59; df = 1; *p* < 0.01^as^;χ^2^ Hosmer-Lemeshow = 59.65; *p* < 0.01^hs^				
Time of exposure to the essential oil				
Constant	−0.295	0.86	−3.43	<0.01^hs^
Time	0.005	0.002	2.91	<0.01^hs^
G = 8.50; df = 1; *p* < 0.01^as^; χ^2^ Hosmer-Lemeshow = 0.937; *p* < 0.01^h^				
Concentration + Time of exposure to the essential oil				
Constant	−1.380	0.113	−12.25	< 0.01^hs^
PPM	0.0026	0.0002	17.35	< 0.01^hs^
Time	0.0059	0.0018	3.32	< 0.01^hs^
G = 341.67; df = 2; *p* < 0.01; χ^2^ Hosmer-Lemeshow = 45.62; *p* < 0.01				
Concentration (PPM) + Time of exposure to the essential oil + Concentration *Time				
Constant	−1.6672	0.1913	−8.71	< 0.01^hs^
PPM	0.0033	0.0004	8.10	< 0.01^hs^
Time	0.0117	0.0036	3.29	< 0.01^hs^
PPM*Time	−0.00001	0.000001	−1.86	0.07^ns^
G = 345.10; df = 3; *p* < 0.01^as^; χ^2^ Hosmer-Lemeshow = 66.55; *p* < 0.01 ns: non-significant; s: significant; hs: highly significant.				

The probit model showed that essential oil concentration and exposure time to essential oil had influenced larval mortality. However, the multiplicative interaction of exposure time and concentration (Table 3, PPM * time) was not significant. For this reason, there was no potentiation of the effect of exposure time on mortality of larvae exposed to the essential oil at the concentrations set.

Despite the highly significant effect of exposure time on larval mortality, the data fitting to the probit model, measured by the *p*-value of χ^2^ of Hosmer and Lemeshow, was much smaller than the effect of the essential oil concentration. When time and concentration were included in the same model, the *p*-value of χ^2^ of Hosmer and Lemeshow tended to converge to the value of concentration of when it was tested separately. The slope associated with the time is much smaller compared to the concentration (Table 3), indicating that the major causative agent of mortality is the essential oil itself.

Nevertheless, the effect of exposure time on larval mortality produced a significant impact on the determination of lethal concentrations. The LC50 decreased in absolute value and converged to a lower confidence interval and with a better fit of χ^2^ of Hosmer and Lemeshow within 72 hours of exposure (Table 3).

The experimental minimum concentration of the essential oil able to induce mortality of all larvae exposed was 862.20 ppm, when we observed that all larvae died about one hour after contact with the essential oil. This experimental value was below the value estimated by the probit model, 1261.239 ppm, which calculated the LC_50_ at 366.9167 ppm at the end of 72 hours of exposure to the essential oil (Table 4). However, the estimated LC50 is within the range of 344.88 - 431.10 μg.mL^−1^ experimentally proposed by Silva et al. [16].

**Table 4:** **Larvicidal activity of lethal concentrations of the essential oil of ripe fruits and seeds of** ***Schinus terebinthifolia*** **Raddi to larvae of** ***Stegomyia aegypti*** **(Linnaeus, 1792) at 24-hour intervals of exposure**

**Time (h)**	**Effect**	**Dose (ppm) ± (95% confidence interval)**
	LC_50_	476.2289 ± 45.9505
	LC_90_	968.8985 ± 71.1853
24	LC_95_	1108.563 ± 82.8280
	LC_99_	1370.552
	Slope	0.0029 ± 0.0005; Z = 10.82; *p* < 0.01^hs^
	Constant	−1.3794 ± 0.2515 Z = −10.72; *p* < 0.01^hs^
	G	132.96; df = 1; *p* < 0.01^hs^
	LC_50_	419. 97 ± 45.5872
	LC_90_	912.6437 ± 67.6247
	LC_95_	1052.309 ± 79.0205
48	LC_99_	1314.297 ± 102.385
	Slope	0.0027 ± 0.00052; Z = 10.22 (*p* < 0.01^hs)^
	Constant	−1.1183 ± 0.2374; Z = −9.33 (*p* < 0.01^hs^)
	G	115.88; df = 1; *p* < 0.01^hs^
	LC_50_	366.9167 ± 45.7382
	LC_90_	859.5862 ± 64.7419
	LC_95_	999.2512 ± 75.8928
72	LC_99_	1261.239 ± 99.027
	Slope	0.0023 ± 0.00052; Z = 10.22 (*p* < 0.01^hs)^
	Constant	−0.8194 ± 0.2231; Z = −7.2 (*p* < 0.01^hs^)
	G	87.83; df = 1; *p* < 0.01^hs^

**hs**: Highly significant.

The LC_50_ determined in our study corresponded to the lowest mean value of 366.9167 ppm, in the period of 72 incubation hours. Cheng et al. [32] demonstrated that the LC_50_ gave the optimal larvicidal activity of essential oils < 100 μg/ml. On the other hand, Pitasawat et al. [33] reported that the inhibitory activity of essential oils has been shown at higher concentrations.

### Morphometric analysis of larvae

**Figure 1:**
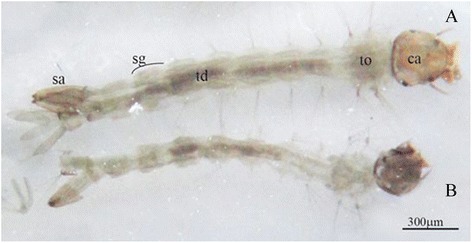
***Stegomyia aegypti*** **.** Third instar larvae exposed to 86.22 ppm **(A)** and 603.24 ppm **(B)** essential oil of *Schinus terebinthifolia* (ca: head; to: thorax; td: digestive tube; sg: segments; sa: air siphon).

Larvae exposed to sub-lethal concentrations of the essential oil showed a greater preservation of the structure of the head, followed by thorax. The eight body segments presented the following alterations: loss of tufts of bristles of segments; reduced thickness of the exoskeleton and loss of integrity of the peritrophic membrane, indicated by shrinkage, and loss of definition observed in the internal organs of the larva (Figure 1).

**Figure 2:**
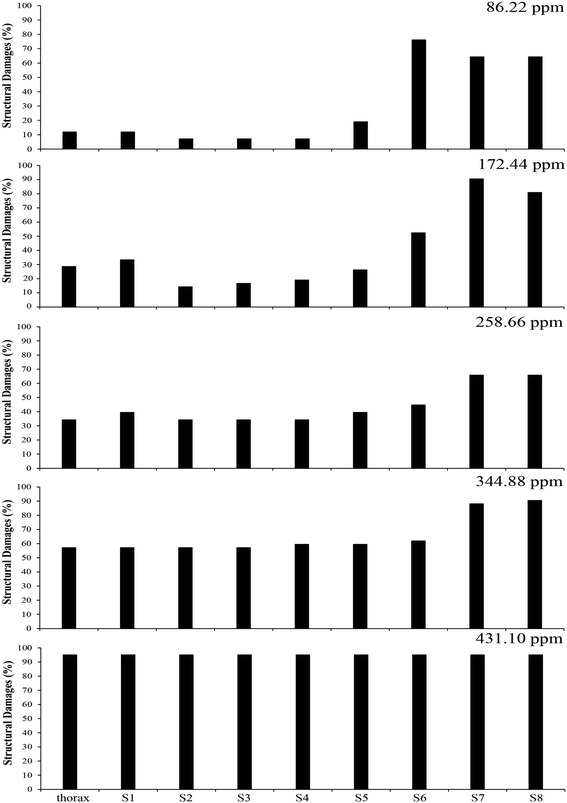
**Loss of structural integrity in thorax and eight body segments of** ***Stegomyia aegypti*** **exposed to the concentration gradient of the essential oil.**

Considering the larval body segmentation, even at concentration of 86.22 ppm, at which over 90% of the exposed larvae survived the treatment (Table 2), the three most distal segments, containing the intestine, Malpighian tubules and air siphon, showed a loss of structural integrity (Figure 2). Thus, it represented the most sensitive region to the effects of the essential oil (Figure 2). After that, structural damages progressed in an acropetal order in the larva. They had begun in median and proximal segments, towards the larvae head. Damages were intensified at the concentration of 344.88 ppm (Figure 2), that was comprised in the 95% confidence interval of the estimated LC_50_ (366.9167 ± 45.7382 ppm, Table 4).

Cases of instantaneous death within one hour after exposure to the essential oil were observed at 862.20 ppm, when the larvae lost the directionality of their upward movements for breath, then died.

**Figure 3:**
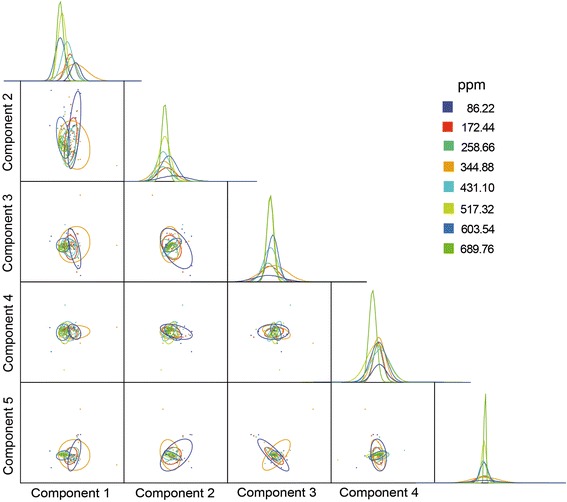
**Diagram of the discriminant analysis performed with morphometric evaluations of** ***Stegomyia aegypti*** **larvae exposed to concentrations of the essential oil, and respective normal dispersion curves.**

The Discriminant Analysis (Figure 3) detected different groups of larvae, concerning their structural damages, in relation to concentrations of the essential oil to which they were exposed (λWilk = 0.423; *p* < 0.01; df: 8; 7; 238). However, the Jackknife cross-validation reached a maximum of 28% accuracy in the classification of the sampled larvae into their respective groups by using the estimated discriminant function, not allowing to accept the groups of larvae as distinct [28] according to the concentrations of the essential oil.

The ordinal logistic regression found a highly significant logistic model (G = 8.18; df = 1; *p* < 0.01). However, the Z standard coefficient of the slope associated with the essential oil concentration was 1.02, which indicates that it had a minor impact on the generation of damage in different segments of the larvae, suggesting that structural damages and death are due to different processes.

Moreover, the instantaneous death of larvae at concentrations greater than or equal to 862.20 ppm, the reduced thickness of the exoskeleton, and the loss of integrity in internal organs of the sixth, seventh and eighth segments of the larval body were the most apparent signs of the effects of the essential oil on *St. aegypti* larvae (Table 5).

**Table 5:** **Percentage of damages on the body segments (S1-8) of the larvae of** ***Stegomyia aegypti*** **(Linnaeus, 1792) that had died within 72 hours of exposure to each concentration of the essential oil of ripe fruits and seeds of** ***Schinus terebinthifolia*** **Raddi**

**Ppm**	**Thorax**	**S1**	**S2**	**S3**	**S4**	**S5**	**S6**	**S7**	**S8**
86.22	11.91	11.91	7.14	7.14	7.14	19.05	76.19	64.29	64.29
172.44	28.57	33.33	14.29	16.67	19.05	26.19	52.38	90.48	80.95
258.66	34.21	39.47	34.21	34.21	34.21	39.47	44.74	65.79	65.79
344.88	57.14	57.14	57.14	57.14	59.52	59.52	61.91	88.10	90.48
431.10	95.00	95.00	95.00	95.00	95.00	95.00	95.00	95.00	95.00
517.32	84.00	84.00	78.00	78.00	78.00	78.00	82.00	92.00	92.00
603.54	78.26	78.26	78.26	78.26	80.43	84.78	84.78	91.30	91.30
689.76	96.00	96.00	92.00	92.00	92.00	94.00	96.00	100.00	98.00

The instant death at toxic concentrations suggests an intervention in a critical process for the larvae. In this case, major damages would be those caused to the Malpighian tubules, since they are responsible for the excretion, not only of electrolytes and metabolites, but also of the high volume of water naturally excreted by insect larvae that develop in water [34].

Nonetheless, at sub-lethal concentration of 86.22 ppm, when a large number of larvae had survived, it is also evident that the tolerance of a toxic effect up to a certain limit, even with a reduced thickness of the exoskeleton and structural damage in the air siphon, Malpighian tubules and intestine. Probably, the damage generated in these structures will gradually be reflected in the loss of structural and functional integrity in other larvae segments of the larva.

Exoskeleton may be involved in the toxicity caused by the essential oil through at least two mechanisms already mentioned in the literature. In one of them, chitin deposition should be preserved and produce the new exoskeleton at the larval stage before the ecdysis which is inhibited, as it had been reported for plant extracts [35]. For instance, azadirachtin, a substance extracted from neen, *Azadirachta indica* A. Juss. (Meliaceae), which acts on neurosecretory system in *St. aegypti*, by blocking the release of ecdysone, increasing its concentration within the *corpus allatum*. The accumulation of ecdysones in *corpus allatum* interferes with the synthesis and release of the prothoracicotropic hormone (PPTH), which is responsible for the production of ecdysone by prothoracic glands [36]. It should not be the case of our study since we observed presence of fourth instar larvae at the concentration of 86.22 ppm after 72 hours of incubation.

The reduced thickness of the exoskeleton, more evident in concentrations near the LC50 (Figure 2), may be related to a decreased chitin synthesis [37] that is a process particularly sensitive to cellular ATP concentrations. The air siphon, intestine and Malpighian tubules, the injured organs, also have cell membranes highly dependent on ATP supply for physiological functioning, involving transport of electrolytes and nutrients [38]. Possibly, the metabolic basis of the damage observed at sub-lethal doses of essential oil is a consequence of a process that decrease cellular ATP supply, most likely promoting the uncoupling of oxidative phosphorylation, since there are reports of death by uncoupling of the respiratory chain in insect larvae [39].

Finally, the results showed a dose-dependent mortality of larvae after exposure to the essential oil of *S. terebinthifolia*. The biological activity of essential oils has been frequently attributed to their major components [29]. However, the participation of other substances present in the essential oil cannot be disregarded, given the possibility of synergism between the compounds [40,41].

## Conclusion

In summary, the essential oil of ripe fruits and seeds of *S. terebinthifolia* causes death to *St. aegypti* larvae after 72 hours exposure, in a dose-dependent manner. It also changes the structure of exposed larvae, evidencing a direct action on the exoskeleton and damage to internal organs of larvae. Our results open up possibilities for the use of natural products as an alternative to the control of dipterans since they are biodegradable and do not affect the environment. Further studies are needed to evaluate the safety of use of this compound on a field scale, seeking to assess environmental security.
